# Family medicine in Tanzania: Seize the moment

**DOI:** 10.4102/phcfm.v12i1.2129

**Published:** 2020-03-10

**Authors:** Riaz Ratanzi, Bernhard M. Gaede

**Affiliations:** 1 Department of Family Medicine, Aga Khan University, Dar-es-Salaam, United Republic of Tanzania; 2 Department of Family Medicine, University of KwaZulu-Natal, Durban, South Africa

**Keywords:** Family medicine, universal health coverage, health system, PHC, Tanzania

## Abstract

In the context of addressing the pressing health needs for the global population, the World Health Organization has repeatedly called for universal health coverage (UHC) to be prioritised by its member countries. This is to be achieved through a high-quality primary health care (PHC) approach that provides comprehensive and integrated generalist care as close to where people live as well as links the clinical care to health promotion and disease prevention. In this paper, we argue for the introduction of family medicines as a critical player in the healthcare system of Tanzania to strengthen the strategies towards UHC. The paper reviews how PHC is understood, the context of family medicine in sub-Saharan Africa and makes a case for how family medicine can assist in addressing the current burden of disease in Tanzania.

The United Nations declaration on universal health coverage (UHC) in September 2019^1^ follows on a long string of calls for strengthening primary health care (PHC) such as the Alma Ata declaration by the World Health Organization (WHO), which celebrated its 40th year with the Astana Declaration in 2018.^2^ Indeed, the Astana Declaration states:

We are convinced that strengthening primary health care (PHC) is the most inclusive, effective and efficient approach to enhance people’s physical and mental health, as well as social well-being, and that PHC is a cornerstone of a sustainable health system for universal health coverage (UHC) and health-related Sustainable Development Goals.^2^ (p. 5)

In many countries throughout the world, family medicine has become central in the pursuit of such a high-quality PHC system and UHC, thereby improving the health of the population.

In sub-Saharan Africa, family medicine is a relatively new discipline and is increasingly playing a central role in the healthcare systems in some countries – most notably Nigeria, South Africa and Ghana.^3^ In the East African region in particular, family medicine has become well-established at the medical universities in Kenya and Uganda and has been incorporated into health system planning.^4,5^ Increasingly, the role of the family physician is recognised in the critical role of advocacy and clinical stewardship that aims for system-wide improvement in the quality of healthcare.^6^ Recent research points to a significant impact that family physicians are making in the overall improvement of health services.^7^ Yet, in some countries, the development of family medicine has lagged behind, and we believe that family medicine has a critical contribution to make in the healthcare system, including in healthcare delivery in Tanzania.

The Tanzania Development Vision 2025 is a commitment to strive for a high quality of life for Tanzanians.^8^ Access to quality primary care for all is a priority for achieving this objective. This is in the context of rapidly increasing incidences of non-communicable diseases and a significant burden of infectious diseases.

In response, the Ministry of Health launched the Primary Health Care Services Development Programme to accelerate the provision of PHC by 2017. Likewise, the overarching theme of the Health Sector Strategic Plan (HSSP IV: July 2015 – June 2020) is access to quality healthcare for all.^9^ Similarly, under the rubric of Big Results Now, the key healthcare results include (1) an increase in human resources for health, (2) availability of healthcare commodities, (3) improvement in health facility performance management and (4) improvement in reproductive, maternal, adolescent and child health.

In all of these key result areas, family physicians can play a substantial role in strengthening integrated PHC in district health services in Tanzania. It is recognised that a people-centred approach^10^ moves beyond the programmatic silos that have characterised health systems approaches in the past. Learning from work conducted under similar settings, the potential roles of family physicians would be:

1. managing complex medical problems associated with chronic diseases and mental health issues that are beyond the capacity of medical officers, assistant medical officers and clinical officers to deal with at the community health centre or at the district hospital level
2. leadership of healthcare teams in clinical governance and quality improvement
3. training and supporting of health personnel, including the categories of health workers, in the district health system
4. collaboration with public health to link clinical care with community-level interventions and approaches.

The care provided by family physicians extends beyond the service provided to individuals at a facility. It addresses the health needs of their families in the context of their social and physical environments. This helps to keep patients out of hospital and lead productive lives. It is critical to note that in the approach to the management of complex clinical problems, direct linkages to improving quality of care, improving healthcare services and strengthening oversight and governance are evident. Indeed, in the recently released Lancet Commission on Quality of Health Care,^11^ it is estimated that a higher number of patients die because of the poor quality of care, than from lack of access to care in lower middle income countries (LMICs). Family physicians by virtue of their expanse of training could play a crucial role in integrating vertical donor-funded programmes into comprehensive and high-quality PHC.

The WHO’s plea to re-orientate PHC was made because the conventional health system models were not living up to the expectation of improving the health of populations in LMICs. A radically different approach is required in the provision of healthcare, which combines health promotion and disease prevention with high-quality primary care.
